# A 3D Organotypic Human Bronchial Model Reveals Persistent Infection and Modulated Inflammatory Response when Exposed to *Brucella abortus*

**DOI:** 10.3390/tropicalmed11030078

**Published:** 2026-03-10

**Authors:** Iván Mathias Alonso Paiva, Florencia Muñoz González, Cecilia Rotondaro, Magali Bialer, Paula Arias, Arlinet Kierbel, Mariana C. Ferrero, Pablo C. Baldi

**Affiliations:** 1 Cátedra de Inmunología, Facultad de Farmacia y Bioquímica, Universidad de Buenos Aires, Buenos Aires 1113, Argentina; ivan_alonsopaiva@yahoo.com.ar (I.M.A.P.); flor.mg@live.com.ar (F.M.G.); 2 Instituto de Estudios de la Inmunidad Humoral (IDEHU), CONICET-Universidad de Buenos Aires, Buenos Aires 1113, Argentina; 3 Centro de Microscopía y Bioimágenes, Fundación Instituto Leloir, IIBBA-CONICET, Buenos Aires 1405, Argentina; crotondaro@leloir.org.ar; 4 Fundación Instituto Leloir, IIBBA-CONICET, Buenos Aires 1405, Argentina; mbialer@leloir.org.ar; 5 Instituto de Investigaciones Biotecnológicas, Universidad Nacional de San Martín (UNSAM)-Consejo Nacional de Investigaciones Científicas y Técnicas (CONICET), San Martín 1650, Argentina; parias@iibintech.com.ar (P.A.); akierbel@iibintech.com.ar (A.K.); 6 Escuela de Bio y Nanotecnologías (EByN), Universidad Nacional de San Martín, San Martín 1650, Argentina

**Keywords:** *Brucella*, bronchial 3D model, bronchial epithelium, lung fibroblasts, cytokines, immune cross-talk

## Abstract

*Brucella* infection is frequently acquired by inhalation, but the pathogen disseminates systemically from the lungs. However, little is known about the interaction of *Brucella* spp. with the airways. Using a 3D air-exposed organotypic human bronchial tissue model (polarized 16HBE14o- bronchial epithelial cells grown over a collagen matrix containing MRC-5 lung fibroblasts), we analyzed *Brucella abortus* replication, translocation and cytokine responses over prolonged post-infection times. Apically inoculated *B. abortus* invaded, replicated and persisted during the whole follow-up (16 days) within the bronchial tissue without inducing cytotoxicity. Viable bacteria were also detected in the conditioned medium (CM) since day five post-infection, indicating release from the basolateral side. In parallel experiments, no invasion or bacterial release was detected for *Escherichia coli*. The levels of IL-6, IL-8 and MCP-1 were increased in CM from *Brucella*-infected 3D cultures and in monocultures of polarized bronchial epithelial cells or lung fibroblasts. Collagenase/gelatinase activity was increased in 3D cultures and MRC-5 monocultures. Infection transference from bronchial cells to lung fibroblasts was documented using monocultures. An immune cross-talk was detected, as cytokine levels were increased in fibroblasts stimulated with bronchial CM, and vice versa. These results suggest that the bronchial mucosa can sustain *B. abortus* persistence, replication and dissemination, and that it induces a proinflammatory response to which both epithelial cells and fibroblasts contribute.

## 1. Introduction

Brucellosis, mainly caused by *Brucella melitensis*, *B. suis*, and *B. abortus*, is a zoonotic disease that affects over 2,000,000 people annually [1]. Inhalation of contaminated aerosols is a frequent route of human infection and has been implicated in outbreaks among slaughterhouse and laboratory workers, as well as rural populations [2,3,4,5]. Furthermore, brucellosis remains the most common laboratory-acquired infection [6]. Due to their high infectivity via the airborne route, *Brucella* species are classified as risk group three agents by NIH. In addition, they are considered potential biological weapons [7] and have been classified as category B bioterrorism agents by the CDC and as biodefense pathogens by NIAID.

Although airborne transmission is epidemiologically relevant, the interaction of *Brucella* with the pulmonary environment remains poorly understood. Several questions remain regarding its ability to persist within human lung tissues, the mechanisms underlying its interaction with respiratory cells, and the local immune responses elicited during infection.

Once inhaled, airborne pathogens, especially those that finally reach the systemic circulation, may first interact with the bronchial epithelium and later with the underlying fibroblastic layer. Beyond functioning as a physical barrier, the bronchial epithelium actively contributes to host defense by secreting cytokines and chemokines that orchestrate the immune response [8,9,10]. Lung fibroblasts, in turn, play a crucial role in modulating inflammation and tissue repair, producing the cytokines [11,12,13] and matrix metalloproteinases (MMPs) involved in extracellular matrix remodeling [14]. Excessive MMP activity during infection can also promote tissue damage and facilitate bacterial dissemination [15].

Since bronchial tissue comprises both epithelial cells and fibroblasts that engage in reciprocal interactions, monocultures of a single cell type are unlikely to capture the full spectrum of host–pathogen dynamics triggered by *Brucella abortus*. Our previous work demonstrated that *B. abortus* can invade and replicate in non-polarized human bronchial epithelial cell lines (16HBE14o- and Calu-6), inducing a modest chemokine response [16,17]. Nonetheless, these monoculture systems often fail to reproduce important structural and functional features of the airway mucosa, such as motile cilia, robust mucus secretion and mature tight-junctional, thereby limiting their translational relevance. It therefore remains unclear whether the bacterium can persist within a fully differentiated bronchial epithelium and modulate epithelial–stromal communication. Although murine respiratory infection models of *Brucella* have demonstrated extended pulmonary colonization [18,19], the fact that the mouse is not a natural host limits the physiological relevance of those findings. Together, these considerations highlight the need for a human bronchial tissue model that more closely approximates the in vivo airway mucosa.

In this study, we used a three-dimensional (3D) air–liquid interface (ALI) model of the human bronchial mucosa composed of epithelial cells (16HBE14o-) cultured on a collagen matrix containing lung fibroblasts (MRC-5) [20,21,22]. This organotypic model better reproduces the physiological structure and cellular interactions of the human airway compared with monocultures, including epithelial stratification, ciliation, mucus production and long-term cell viability, and has been used to evaluate the interaction of some respiratory pathogens with the bronchial tissue. While this model lacks immune cells, it allows an evaluation of the early interaction of respiratory pathogens with the main structural components of this tissue and has been used to model other respiratory infections [22,23,24]. Using this model, we investigated infection and long-term persistence of *B. abortus* in the bronchial mucosa, and the local innate immune response elicited by the pathogen. We also examined the contribution of fibroblasts to the infectious process and innate immunity, and their interaction with bronchial epithelial cells.

## 2. Materials and Methods

### 2.1. Bacterial Strains and Growth Conditions

*Brucella abortus* 2308, *B. abortus*–GFP, and the *B. abortus* 2308 Δ*virB10* mutant were obtained from Dr. Diego Comerci, and the procedures for the construction of the last two can be found elsewhere. The Δ*virB10* mutant lacks a component of the Type IV secretion system of *Brucella* that is essential for its intracellular survival in several infection models. All the three strains exhibit a smooth phenotype. Bacteria were grown overnight in tryptic soy broth (TSB); for the GFP-expressing strain, kanamycin (50 µg/mL; Gibco, Thermo Fisher Scientific, Waltham, MA, USA) was added to maintain plasmid selection. Cultures were harvested by centrifugation (6000 × *g*, 15 min) and washed twice with phosphate-buffered saline (PBS) prior to infection. All procedures involving live *Brucella* were conducted in biosafety level 3 facilities.

### 2.2. Cell Lines and Growth Conditions

The 16HBE14o- human bronchial epithelial cell line (kindly provided by Dr. Dieter Gruenert), established from normal bronchial epithelium [25], was routinely grown in fibronectin/collagen/BSA-coated flasks in minimal essential medium (MEM; Gibco, Thermo Fisher Scientific, Waltham, MA, USA) supplemented with 10% fetal calf serum (FCS; Natocor, Argentina), 2 mM L-glutamine, 100 U/mL penicillin, and 100 mg/mL streptomycin (all from Gibco, Thermo Fisher Scientific, Waltham, MA, USA). The human lung fibroblast MRC-5 cell line (ATCC CCL-171), which was derived from normal lung tissue of a 14-week-old male fetus, was maintained in supplemented MEM.

### 2.3. Construction of the 3D Bronchial Model

A 3D model of the human bronchial mucosa was constructed in vitro, as described by Nguyen Hoang et al. [20]. Briefly, the inner compartment of Transwell inserts (0.33 µm pore size; Costar, Corning Inc., Corning, NY, USA) was incubated for 30 min at 37 °C with a solution of collagen type I (Gibco, Thermo Fisher Scientific, Waltham, MA, USA, 1.1 mg/mL in MEM medium) to allow collagen coating. A suspension of human lung fibroblasts (MRC-5 cell line; ATCC, Manassas, VA, USA) was added at 2 × 10^4^ cells/Transwell in MEM containing 10% FCS and 1.1 mg/mL type I collagen and incubated for 2 h at 37 °C. After collagen polymerization, 600 µL of MEM containing 10% FCS was added to the basolateral compartment of the Transwell insert. The culture was incubated for 7 days, with medium exchange in both compartments every other day, to allow the growth of fibroblasts embedded in the collagen matrix. Under these conditions, the number of fibroblasts remains stable as the matrix restricts their proliferation [20]. Then, 16HBE14o- bronchial epithelial cells were added on top of the collagen matrix at 3.3 × 10^4^ cells/Transwell and were grown under liquid–liquid conditions for 3 days. Afterwards, the 3D models were kept under air–liquid conditions (ALI) for a further 10 days to allow the polarization of epithelial cells. At this point the 3D models were either infected with *B. abortus* or fixed with 4% paraformaldehyde for histological studies. In the latter case the fixed models were included in paraffin, cut into 5 µm thick sections and stained with hematoxylin-eosin. All cell cultures (either individual cell types or the 3D model) were kept in water-jacketed incubators at 37 °C in a 5% CO_2_ atmosphere.

### 2.4. Infection of the 3D Bronchial Model

The 3D bronchial models were infected from the apical side (bronchial epithelial cells) with *B. abortus* 2308 at 1 × 10^7^ colony-forming units (CFU) per Transwell in a final volume of 10 µL. This inoculum provided a multiplicity of infection (MOI) of around 300, which is well within the range of MOI used in studies on *Brucella* infection of epithelial cells. Two hours later (time 0 post-infection, p.i.) the 3D models were incubated for 1 h with MEM-10% FCS supplemented with 100 µg/mL gentamicin and 50 µg/mL streptomycin (MEM-GS) in both compartments to kill extracellular bacteria. After this time, the culture medium was replaced with MEM-10% FCS without antibiotics and the culture continued in ALI. after . Infected cultures were followed until 16 days p.i., and during this period the cultures were transferred every two days to new wells of the plate containing fresh medium. At different times p.i., the basolateral conditioned medium (CM) from three infected cultures chosen at random was harvested. An aliquot of CM was plated on TSA to allow us to perform CFU counting, while additional aliquots were kept frozen until measurement of cytokine levels, lactate dehydrogenase (LDH) activity, and collagenase/gelatinase (MMPs) activity could be undertaken. In addition, the cells from the same 3D models processed at each time point were lysed with 0.2% Triton X-100 in miliQ water to enumerate intracellular CFU.

At the same time, other 3D cultures were infected with the non-invasive *Escherichia coli* HB101 strain following the same infection protocol used for *Brucella abortus*. This strain was included as a control to rule out any process related to the procedure or the model itself that could allow extracellular bacterial development. To this end, basolateral conditioned medium was collected immediately after infection and at later time points and plated for CFU enumeration. In parallel, CFU were also determined in the tissue lysates at the indicated time points, as described above. The detection limit of the assay was 10 CFU.

### 2.5. Immunofluorescence of the 3D Bronchial Model

The 3D model of bronchial tissue infected with *B. abortus*–GFP was fixed with 4% paraformaldehyde (PFA) for 45 min. After removing the fixative, the samples were washed with PBS and incubated in a 30% sucrose solution overnight at 4 °C to ensure proper cryoprotection. For embedding, the CRYOPLAST freezing medium (Biopack, code 1204.05) was used. Each block included the 3D model together with the porous membrane, which was trimmed during block preparation. The blocks were rapidly frozen in liquid nitrogen and stored at −20 °C until processing. Sections were obtained using a Leica Ag Protect cryostat, generating 10 µm slices that were subsequently mounted on gelatin-coated slides. Immunofluorescence staining of tissue sections was performed, as previously described [20]. E-cadherin was detected using a mouse monoclonal antibody (ab75055, 1:100; Abcam, Cambridge, UK), incubated overnight at 4 °C. The following day, the sections were washed with PBS-Tween and incubated for 1 h at room temperature with a Cy3-conjugated anti-mouse secondary antibody (715-165-150, 1:200; Jackson ImmunoResearch, West Grove, PA, USA). After further PBS washes, cell nuclei were counterstained with 4′,6-diamidino-2-phenylindole (DAPI, Sigma-Aldrich, St. Louis, MO, USA). Images were acquired on a Zeiss LSM 710 confocal microscope equipped with a Plan-Apochromat 40×/1.3 NA objective (Carl Zeiss, Jena, Germany).

### 2.6. Infection of Polarized Bronchial Epithelial Cells

The upper surface of the membrane of Transwell inserts (3 µm pore diameter) was coated with a solution of type I collagen (1.1 mg/mL in MEM medium) and fibronectin for 30 min at 37 °C. 16HBE14o- cells were then cultured at a density of 1 × 10^5^ cells/cm^2^ for 10–14 days under liquid–liquid conditions to allow formation of a confluent monolayer of polarized cells with tight junctions. After this time, the epithelia were kept under ALI conditions for another 10 days to allow complete polarization of the epithelial cells [26]. Cells were apically infected with *B. abortus* 2308 (1 × 10^7^ CFU/Transwell). Two hours later (time 0 p.i.) the monolayers were incubated for 1 h with MEM-GS. Then, the culture medium was replaced with MEM 10% FCS without antibiotics. At different times p.i., an aliquot of the basolateral CM was harvested and kept frozen until measurement of cytokine levels and LDH activity could be undertaken. In addition, the bronchial epithelial cells were lysed with 0.2% Triton X-100 in miliQ water, and the lysates were plated on TSA to enumerate CFU.

### 2.7. Infection of the MRC-5 Lung Fibroblasts

Human lung fibroblasts (MRC-5 cell line) grown in 24-well culture plates were infected with *B. abortus* 2308 at different MOI for 2 h. Cells were washed with sterile PBS to eliminate unbound bacteria and were incubated for 1 h with MEM-GS. At the end of this incubation, the cells were washed three times with PBS, and MEM without antibiotics was added. At 2, 24 or 48 h p.i., the infected cells were lysed for CFU counting. Culture supernatants were harvested and kept frozen at −80 °C for measuring cytokines, LDH activity (cytotoxicity) and MMPs activity.

### 2.8. Cytokine and Chemokine Measurement

Human IL-6, CXCL-8 (IL-8) and CCL2 (MCP-1) were quantified in culture supernatants of 3D models or cellular monocultures by sandwich ELISA (all from BD Biosciences, San Jose, California, USA) using paired cytokine-specific monoclonal antibodies, according to the manufacturer’s instructions.

### 2.9. Evaluation of Gelatinase/Collagenase (MMP) Activity

MMPs activity in the CM from the 3D model and from MRC-5 cells’ infection was measured using EnzChek Gelatinase/Collagenase Assay Kit (Molecular Probes, Invitrogen, Carlsbad, CA, USA). This kit contains gelatin so heavily labeled with fluorescein that fluorescence is quenched. The release of fluorescent peptides as a result of gelatinase activity produces a fluorescence increase which is proportional to the proteolytic activity. Briefly, CM were incubated for 24 h with the fluorescein-gelatin conjugate and the resulting fluorescence intensity was measured with a Victor3 microplate spectrofluorometer (PerkinElmer, Waltham, MA, USA).

### 2.10. Cytotoxicity Assay

To evaluate whether *B. abortus* infection produces a cytotoxic effect, the release of LDH to the culture medium was determined using CytoTox 96 non-radioactive cytotoxicity assay (Promega, Madison, WI, USA). Results are expressed in arbitrary units.

### 2.11. Signaling Pathways

To determine the signaling pathways involved in cytokines and chemokines production, MRC-5 cells were pretreated with BAY 11-7082 (NF-κB inhibitor), LY294002 (PI3-kinase inhibitor), SB203580 (p38 MAPK inhibitor) or DMSO (used as vehicle) (all from Sigma-Aldrich, St. Louis, MO, USA) 1 h before infection with *B. abortus* and were kept throughout the experiment. At 24 h p.i., culture supernatants were harvested and kept frozen at −80 °C for measuring cytokines and chemokines.

### 2.12. Stimulation with Conditioned Media

A quantity of 16HBE14o- human bronchial epithelial cells were infected with *B. abortus* 2308 at MOI of 200 for 2 h. Cells were washed with sterile PBS to eliminate unbound bacteria and were then incubated with MEM-GS. Conditioned media from infected bronchial epithelial cells (INF CM) were harvested 48 h p.i., sterilized by filtration and used to stimulate lung fibroblasts. Conditioned media from non-infected cells (NI CM) were used as controls. The supernatants of the stimulated cultures were collected at 24 h post-stimulation and stored until the cytokine content was measured. The same stimulation procedure was performed in reverse, i.e., stimulating bronchial epithelial cells with CM from infected or non-infected fibroblasts.

### 2.13. Infection Transference from Bronchial Epithelial Cells to Lung Fibroblasts

Bronchial epithelial cells (16HBE14o-) were seeded on coverslips (1.5 cells/well) in well plates in culture medium without antibiotics. Cells were infected with *B. abortus*-GFP at a MOI of 500 bacteria/cell. Cells were centrifuged at 400 × *g* for 15 min and incubated for 2 h at 37 °C in a 5% CO_2_ atmosphere. Then, cells were incubated in medium containing 100 ug/mL gentamicin for 24 h to kill extracellular bacteria. Cells were then washed with sterile PBS and 1 × 10^4^ MRC-5 cells were added to each well in culture medium without antibiotics. The coculture was kept under these conditions for 72 h to allow bacteria egressing from bronchial cells to infect lung fibroblasts. Cocultures were washed and cells were fixed with 4% paraformaldehyde for 1 h, washed again with sterile PBS, permeabilized with 0.3% Triton X-100 for 15 min, blocked with 2% BSA (Sigma-Aldrich, St. Louis, MO, USA) in PBS for 45 min, and stained with phalloidin for 1 h. Images were obtained in the XY plane along the Z axis with a confocal laser-scanning microscope Olympus FV1000 using a PlanApo N (60X 1.42 NA) oil objective (Olympus, Tokyo, Japan). Images were processed with the IMageJ v1.53 software (NIH, Bethesda, MD, USA) and the ZProject tool was used. Cell types were identified by their typical morphology.

### 2.14. Statistical Analysis

Results were analyzed after confirming normal distribution (Shapiro–Wilk test) and homogeneity of variances (Levene’s test or Bartlett’s test, as appropriate). Once assumptions were verified, data were subjected to an unpaired *t*-test or one-way ANOVA followed by Tukey’s or Dunnett’s post hoc test (GraphPad Prism 6.0). All tests were performed at least three times in triplicates. Values are presented as mean ± SD.

## 3. Results

### 3.1. B. abortus Infects, Replicates and Persists in a 3D Model of Bronchial Mucosa

**Figure 1:**
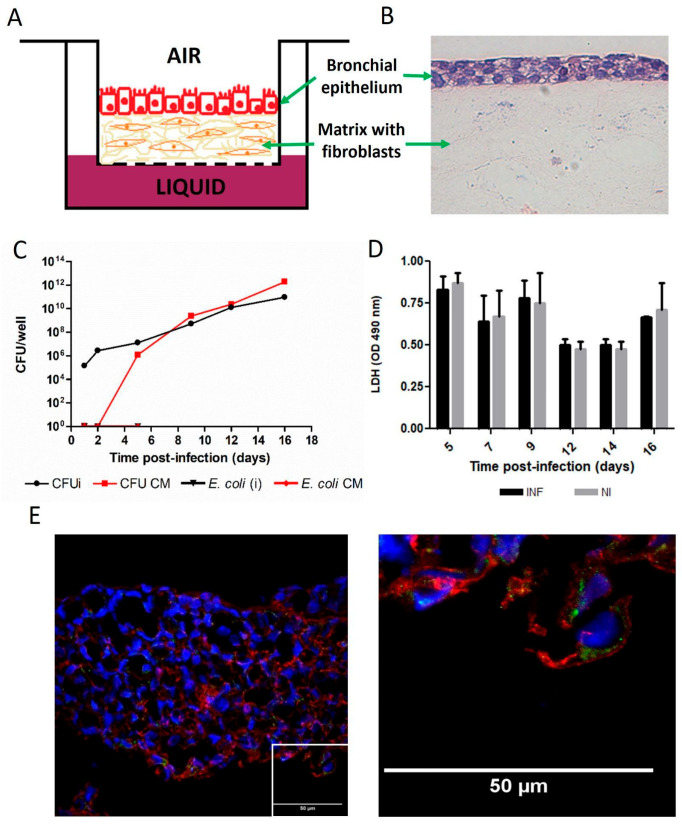
***B. abortus*** infects, replicates, and translocates in a 3D model of human bronchial mucosa without inducing cytotoxicity. (**A**) Schematic representation of the bronchial models constructed in Transwell inserts. (**B**) Hematoxylin and eosin staining of the 3D model where the epithelial layer and the underlying fibroblastic layer embedded in collagen are observed. (**C**) The 3D models were infected for 2 h and then treated for 1 h with gentamicin. After incubation, medium was replaced by culture medium without antibiotics and colony-forming units (CFU) were determined at different days post-infection in the bronchial tissue (intracellular CFU, CFUi) and in the basolateral conditioned medium (CFU CM). Parallel cultures infected with *E. coli* using the same protocol were observed in the same way for 5 days. (**D**) LDH release was measured in CM. Data shown correspond to one representative experiment of three performed with similar results. Values express mean ± SD. (**E**) The left panel shows a representative cryosection (8 µm) of the bronchial tissue model infected with *B. abortus*-GFP at day 5 p.i., revealing epithelial architecture and bacterial localization. E-cadherin (red) delineates the epithelial cell borders, GFP fluorescence (green) identifies intracellular *B. abortus*, and DAPI (blue) labels cell nuclei. Images were acquired on a Zeiss LSM 710 microscope equipped with a Plan-Apochromat 40×/1.3 NA objective. The right panel shows a detail of the inset, in which bacteria are clearly observed inside epithelial cells.

A 3D model of the human bronchial mucosa was constructed in Transwell inserts, as shown in Figure 1A [20]. Polarized human bronchial epithelial cells (16HBE14o) were grown on top of a collagen matrix containing human lung fibroblasts (MRC-5 cell line), and the coculture was maintained under ALI conditions (Figure 1B). As shown in Figure 1C, *B. abortus* was able to replicate within the 3D bronchial model as early as 48 h post-infection, and the intracellular bacterial (CFUi) load continued to increase up to 16 days post-infection, indicating the establishment of a persistent infection. Remarkably, this prolonged presence occurred without evidence of cytotoxicity, underscoring the ability of *B. abortus* to survive and multiply within the bronchial tissue without causing overt damage to host cells (Figure 1D). In contrast, in parallel experiments performed with non-invasive *E. coli*, no bacteria were detected inside the 3D model, which is consistent with its extracellular nature.

Notably, viable *B. abortus* were detected in the basolateral culture medium (CM) from day five post-infection onwards, indicating release of bacteria from the infected 3D bronchial tissue. In parallel experiments performed with the non-invasive bacterium *E. coli* HB101, no CFU were detected in the basolateral compartment at early time points (up to five days p.i., Figure 1). The absence of this non-invasive control suggests that the presence of *B. abortus* in the bronchial tissue model and later in the basolateral CM is related to its capacity to invade (and exit) cells and not to a technical artifact.

Analysis of cryosections from the 3D airway construct at five days p.i. revealed that *B. abortus* predominantly infected the epithelial compartment. Confocal imaging showed GFP-expressing bacteria localized within the E-cadherin–positive bronchial epithelial layer (Figure 1E), indicating that the epithelium constitutes the main site of bacterial replication in this model. A comparison against a control non-infected tissue (Supplementary Figure S1) indicates that *B. abortus* infection did not affect the overall architecture of the 3D model.

### 3.2. B. abortus Infection Induces a Proinflammatory Response in the 3D Model of Bronchial Mucosa

**Figure 2:**
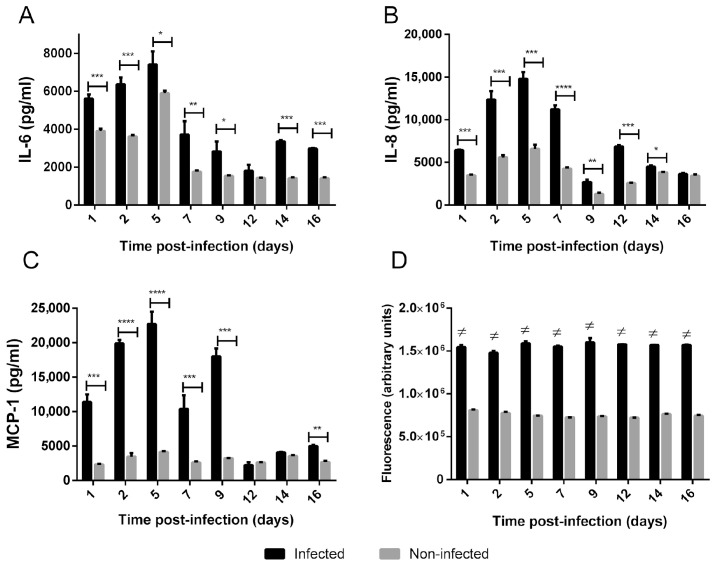
**Secretion of cytokines, chemokines and MMPs by the 3D bronchial model infected with*****B. abortus***. CM were harvested at different days p.i. (**A**–**C**) IL-6, IL-8 and MCP-1 concentrations in CM were measured by ELISA. Data express means ± SD. Asterisks indicate significant differences (*, *p* < 0.05; **, *p* < 0.01; ***, *p* < 0.001; ****, and *p* < 0.0001) vs. non-infected controls (Student’s *t*-test). (**D**) Gelatinase/collagenase activity was determined by EnzCheck assay. Data express means ± SD. #, *p* < 0.0001 vs. non-infected controls (Student’s *t*-test).

To evaluate the innate immune response of the 3D culture to *B. abortus* infection, the levels of certain cytokines and chemokines, previously shown to be induced early by *Brucella* in several non-phagocytic cells, were measured in the basolateral CM at different p.i. times. As shown in Figure 2A–C, the levels of IL-6, IL-8 and MCP-1 were increased in CM from infected 3D cultures as compared to non-infected cultures at several p.i. times during follow-up. In addition, an increased level of MMP activity was detected in the CM from infected cultures as compared to non-infected controls at all time points (Figure 2D).

### 3.3. B. abortus Infects Polarized Bronchial Epithelial Cells and Induces a Proinflammatory Response

**Figure 3:**
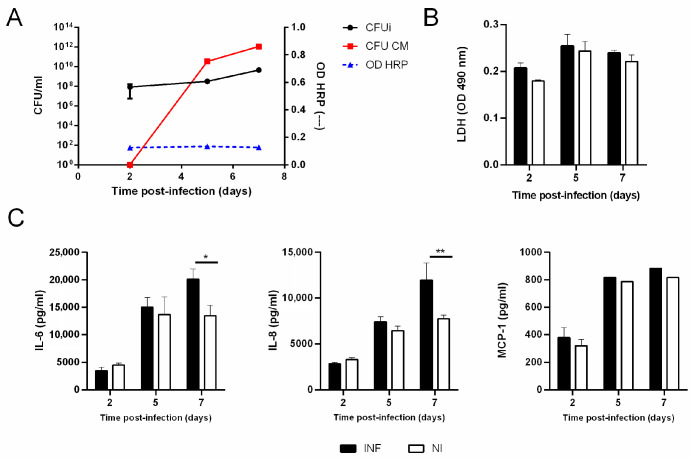
***Brucella abortus*** infects and elicits a delayed proinflammatory response in polarized bronchial epithelial monolayers. Confluent 16HBE14o- cells were grown on Transwell inserts for 14 days under liquid–liquid conditions and subsequently maintained under ALI conditions for an additional 10 days to achieve full epithelial polarization. Monolayers were then apically infected with *B. abortus* for 2 h. After incubation, the inoculum was removed and replaced with antibiotic-free culture medium. (**A**) Intracellular bacterial load in the bronchial monolayers (CFU_i_), bacterial counts in the basolateral conditioned medium (CFU CM), and peroxidase activity (HRP OD) were assessed at different days post-infection. (**B**) LDH activity was measured in basolateral conditioned media from infected (INF) and non-infected (NI) cultures. (**C**) Levels of IL-6, IL-8, and MCP-1 in the basolateral compartments of INF and NI cultures were quantified by ELISA at the indicated time points. Data represent means ± SD. Asterisks denote statistically significant differences (* *p* < 0.05 and ** *p* < 0.01) relative to non-infected controls (ANOVA followed by Tukey’s test). Values correspond to three independent experiments.

To strengthen our findings from the 3D airway model, we next examined infection dynamics and cytokine responses in polarized 16HBE14o- monolayers cultured under ALI conditions, a 2D reductionist system widely used to study epithelial interactions with respiratory pathogens [26]. As shown in Figure 3A, *B. abortus* infected and replicated intracellularly in the monolayers from day two to day seven post-infection (p.i.). No bacteria were detected in the basolateral compartment at day two p.i., but CFU became detectable from day five onward and increased by approximately two logs by day seven. HRP activity and LDH release in basolateral supernatants remained unchanged throughout the experiment (Figure 3A,B), indicating that epithelial integrity and barrier function were preserved. Notably, the timing of basolateral translocation in this ALI monolayer model mirrored that observed in the 3D bronchial epithelial model (Figure 1C), in which *B. abortus* also crossed the epithelial barrier only at late p.i. time points.

Together, these results show that *B. abortus* undergoes late post-infection transcellular passage across a polarized bronchial epithelial monolayer without compromising barrier integrity.

In parallel, infection of polarized monolayers induced a delayed proinflammatory response. As shown in Figure 3C, the infection induced an increased secretion of IL-6 and IL-8 at day five p.i., which became significant by day seven p.i. In contrast, no significant increase in MCP-1 was detected in the basolateral compartment during the follow-up period.

### 3.4. B. abortus Released from Bronchial Epithelial Cells Can Infect Lung Fibroblasts

Non-lytic egress via autophagic-like vacuoles enables *Brucella* to exit infected cells and disseminate to neighboring cell populations [27]. Having observed in both the 3D airway model and the ALI monolayers that *B. abortus* undergoes late basolateral translocation, we next assessed whether bacteria released from infected bronchial epithelial cells could infect lung fibroblasts.

**Figure 4:**
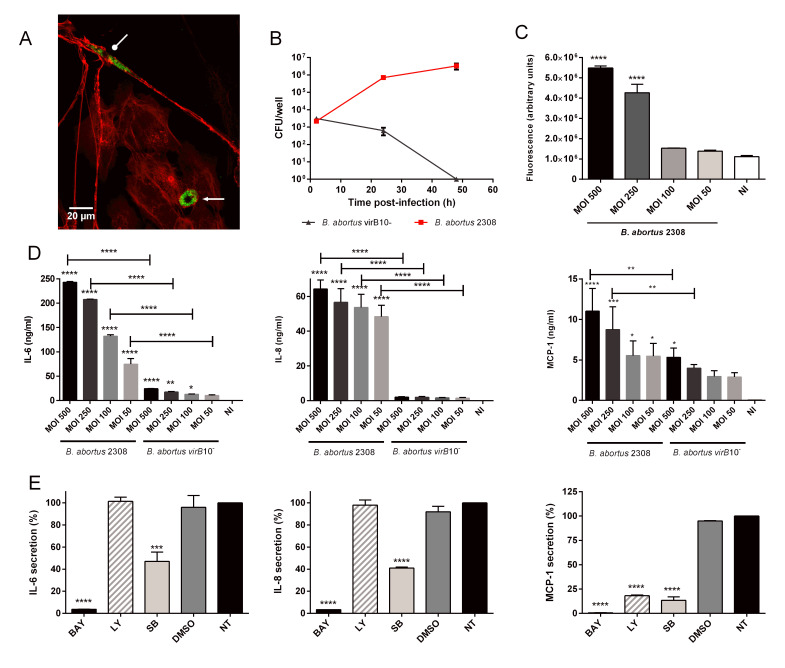
***B. abortus*****infects and induces an innate immune response in human lung fibroblast**. (**A**) Bronchial 16HBE14o- cells were infected for 2 h with *B. abortus*-GFP and later treated with gentamicin for 24 h. At this point, MRC-5 lung fibroblasts were added in culture medium without antibiotics. After 72 h of incubation the coculture was stained with phalloidin and analyzed by confocal microscopy (60×). A projected Z stack is shown. Bronchial epithelial cells (pointed arrow) and fibroblasts (rounded arrow) were identified by morphology. Green: *B. abortus*-GFP and red: phalloidin. (**B**) MRC-5 cells were infected for 2 h with *B. abortus* or *B. abortus virB10-* (MOI 250) and CFU were measured at different times p.i. Values express mean ± SD. (**C**) Conditioned media were harvested 24 h p.i. and the collagenase/gelatinase activity was assessed using the EnzChek commercial kit. Data express means ± SD. Asterisks indicate significant differences (****, *p* < 0.0001) with data from non-infected controls (NI) (ANOVA followed by Dunnett’s test). (**D**) IL-6, IL-8 and MCP-1 concentrations were measured by ELISA in conditioned media harvested at 24 h p.i. Data express means ± SD. Asterisks indicate significant differences (*, *p* < 0.05; **, *p* < 0.01; ***, *p* < 0.001; ****, and *p* < 0.0001) vs. non-infected (NI) controls or between cells infected with different strains at the same MOI. (**E**) Cells were treated or not (INF) with BAY 11-7082 (BAY, NF-κB inhibitor), LY294002 (LY, PI3-kinase inhibitor), SB203580 (SB, p38 MAPK inhibitor), or vehicle (DMSO) for 1 h before infection with *B. abortus.* The inhibitors were kept throughout the experiment. At 24 h p.i., culture supernatants were harvested for measuring IL-6, IL-8 and MCP-1 by ELISA. The inhibition percentage was calculated taking as reference the secretion of each cytokine by INF cells (100%). Data express means ± SD. Asterisks indicate significant differences (***, *p* < 0.001; ****, and *p* < 0.0001) vs. INF controls (ANOVA followed Tukey test).

To this end, 16HBE14o- cells were infected with *B. abortus*-GFP, incubated for 24 h in the presence of antibiotics to eliminate extracellular bacteria, and subsequently cocultured with uninfected MRC-5 fibroblasts for 72 h. Confocal microscopy of phalloidin-stained cocultures revealed fibroblasts harboring numerous GFP-expressing bacteria (Figure 4A). These observations demonstrate that viable *B. abortus* released from infected epithelial cells are able to infect lung fibroblasts, supporting the potential role of the latter as secondary targets during bronchial infection.

### 3.5. B. abortus Infects and Replicates in Human Lung Fibroblasts

Because fibroblasts became infected in the coculture system shown in the previous section, we next assessed their intrinsic ability to support *B. abortus* replication by infecting MRC-5 cells in a monoculture. As shown in Figure 4B, *B. abortus* invaded and replicated in MRC-5 cells grown in 24-well culture plates. CFUs increased by two logs during the first 24 h and a further log at 48 h p.i. It has been shown in many cell types that *B. abortus’* ability to survive and replicate intracellularly depends on the expression and function of the type IV secretion system encoded by the virB genes [16,28,29]. In agreement with those previous studies, a virB10- mutant was unable to replicate or survive in MRC-5 cells (Figure 4B).

### 3.6. B. abortus Infection Induces a Proinflammatory Response in Human Lung Fibroblasts

After confirming that *B. abortus* replicates efficiently in MRC-5 fibroblasts, we assessed the inflammatory responses elicited by infection. Cytokines, chemokines, and MMP activity were measured in the conditioned medium of infected cultures. As shown in Figure 4C, a significant increase in MMP activity was observed in the CM of lung fibroblasts infected with the two highest MOI. As shown in Figure 4D, the infection induced, in a MOI-dependent manner, a significant increase in the levels of IL-6, IL-8 and MCP-1 in the CM of infected MRC-5 cells as compared to uninfected cells. Whereas infection with the virB10 mutant also induced a significant increase in IL-6, the levels attained were markedly lower than those induced by the wild-type strain, likely reflecting the reduced intracellular bacterial load. To investigate the signaling pathways involved in these cytokine responses, cells were pretreated with specific inhibitors before infection. As shown in Figure 4E, IL-6 and IL-8 responses were inhibited by BAY 11-7082 and SB203580, indicating the involvement of NF-κB and MAPK p38 pathways. MCP-1 responses were inhibited by all three inhibitors, indicating that, in addition to these pathways, the PI3K pathway was also involved.

### 3.7. Intercellular Communication Between Epithelial Cells and Fibroblasts Amplifies Cytokine Production

In both the in vivo lung environment and in the 3D model used here, bronchial epithelial cells and lung fibroblasts reside in close proximity and can engage in bidirectional communication through soluble mediators. During *B. abortus* infection, these reciprocal interactions may shape the overall cytokine milieu beyond the individual responses elicited by each cell type, as described above. Thus, the cytokine profile observed in the 3D model likely reflects not only the intrinsic responses of fibroblasts and epithelial cells, but also the additional stimulation that each cell type receives from infection-induced factors released by the other. To assess these cross-talk mechanisms, we stimulated each cell type with conditioned media (CM) from infected or non-infected cultures of the counterpart cell type.

**Figure 5:**
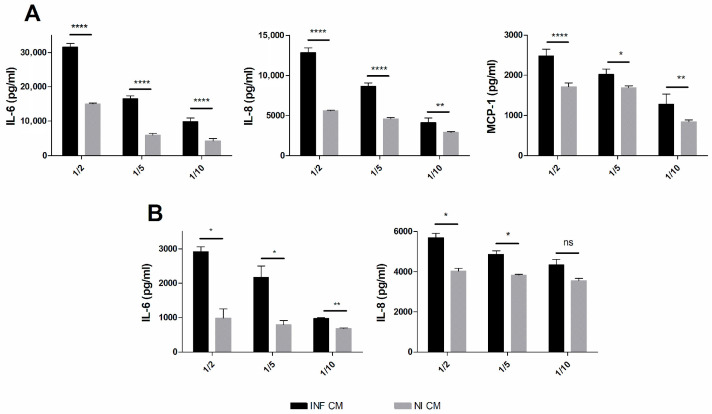
**Cytokine cross-talk between lung fibroblasts and bronchial epithelial cells.** MRC-5 fibroblasts were stimulated with conditioned media (CM) from *B. abortus*-infected bronchial epithelial cells (**A**) or bronchial epithelial cells were stimulated with CM from *B. abortus*-infected MRC-5 fibroblasts (**B**). In each panel, INF CM indicates stimulation with CM from infected cells, whereas NI CM indicates stimulation with CM from non-infected cells used as controls. The cells were exposed for 24 h to different dilutions of INF CM and NI CM and the concentrations of IL-6, IL-8 and MCP-1 were quantified by ELISA. Data express means ± SD of cytokine levels produced specifically by each cell type, from which the concentrations already present in the INF CM or NI CM used for stimulation were subtracted. Asterisks indicate significant differences (*, *p* < 0.05; **, *p* < 0.01; ****, *p* < 0.0001; ns, non-significant) with non-infected controls (Student’s *t*-test).

As shown in Figure 5A, the levels of all three cytokines evaluated were significantly increased in fibroblasts stimulated with CM from *B. abortus*-infected bronchial epithelial cells as compared to those stimulated with CM from non-infected bronchial cells. Similarly, cytokine levels were significantly increased in bronchial epithelial cells stimulated with CM from infected fibroblasts as compared to those stimulated with CM from non-infected fibroblasts (Figure 5B). These results show that lung fibroblasts and bronchial epithelial cells can produce proinflammatory mediators as a result of reciprocal interactions through factors secreted by each cell type in response to *B. abortus* infection.

## 4. Discussion

The respiratory tract is one of the most common routes exploited by species of the genus *Brucella* to gain entry to the organism and, thus, be able to reach the systemic circulation. *B. abortus* is known to infect human respiratory epithelial cells, but the relevance of these observations has been limited by the use of non-polarized monocultures that do not reproduce the structural and functional features of the airway mucosa [16].

Fibroblasts actively interact with the epithelial layer and play a key role in shaping local inflammation through the secretion of cytokines [11,12]. Therefore, one of the most physiologically relevant in vitro approaches to study pathogen–bronchial tissue interactions is the 3-dimensional (3D) air-exposed organotypic model of the bronchial mucosa, in which differentiated bronchial epithelial cells are grown atop a collagen matrix containing fibroblasts [20]. This well-established system has been used to investigate early host–pathogen interactions during *Mycobacterium tuberculosis* infection [21] and staphylococcal pneumonia [22]. In these models, epithelial and stromal cells self-organize into a structure closely resembling the human airway mucosa, including multiple layers of stratified and ciliated epithelial cells, mucus secretion, and long-term viability. Importantly, these features make the model particularly suitable for studying the persistence of *Brucella* in the lung and its potential role as a pulmonary reservoir for dissemination as previous murine studies have shown that the bacterium can persist in the lungs for several weeks following respiratory infection [30].

In the present study, we implemented this 3D human bronchial mucosa model to evaluate how *B. abortus* interacts with, persists within, and potentially spreads from the airway tissue. All the major *Brucella* species, including *B. abortus*, are known to infect humans through the respiratory route [6,31]. Given the complexity of the 3D model, we focused our analyses on *B. abortus*, which has been extensively used in in vitro models of *Brucella* infection.

Our study shows that *B. abortus* infects and persists for extended periods within the organotypic bronchial tissue model, as reflected by the sustained intracellular bacterial load (CFU_i_) up to 16 days p.i., without inducing detectable cytotoxicity. Consistent with these quantitative findings, fluorescence imaging of GFP-expressing *B. abortus* revealed intracellular bacteria within the epithelial layer at five days p.i., further supporting the notion that the epithelium serves as a long-term intracellular niche. Importantly, this pattern of long-term intracellular persistence accompanied by the release of viable bacteria to the basolateral compartment (CFU CM) is consistent with the behavior observed in polarized 2D epithelial monolayers. This concordance reinforces the notion that *Brucella* persistence and non-lytic egress are intrinsic features of the respiratory infection cycle and are not dependent on the structural complexity of the respiratory model but rather on the biology of the pathogen–host interaction.

A similar profile of prolonged intracellular survival with basolateral dissemination has been reported for Andes hantavirus in the same 3D bronchial mucosa system, with viral progeny detected up to day 25 p.i. [32]. The ability of *B. abortus* to establish a persistent replication niche in the lung mucosa from which viable bacteria are steadily released may contribute to its capacity to disseminate systemically from an initial respiratory portal of entry [18]. Studies performed in *Macaca mulatta* demonstrated the ability of *Brucella* to disseminate systemically after airborne transmission [33,34,35,36,37]. These studies show that, after entering the airways, *Brucella* reaches the systemic circulation, implying the existence of one or more mechanisms for the systemic release of *Brucella* from the infected airways. Our findings regarding the prolonged release of *B. abortus* to the basolateral compartment of the 3D bronchial model are in line with these observations.

Notably, the absence of cytotoxicity in our model aligns with the typically mild or clinically silent pulmonary manifestations observed in human brucellosis despite inhalational exposure [18].

Recent work is beginning to reveal the mechanisms by which *B. abortus* exits host cells without inducing lysis, a process historically underexplored. Live-cell imaging studies demonstrated that *B. abortus* co-opts the multivesicular body (MVB) pathway to exit host cells: bacteria are released in CD63^+^/LBPA^+^ vesicles, and pharmacological modulation of MVB biogenesis alters egress efficiency [38]. A complementary mechanism has also been described in which *B. abortus* induces mitochondrial fragmentation and BNIP3L-dependent mitophagy in HeLa cells, leading to the formation of mitochondria-derived *Brucella*-containing vacuoles (mBCVs) that mediate late-stage egress. Consistently, BNIP3L depletion severely impairs bacterial release and reinfection [39]. Together, these findings indicate that *Brucella* displays mechanistic plasticity in its egress pathways, engaging distinct vesicular trafficking routes (MVB and mBCVs), likely influenced by host cell type, metabolic state, and tissue context. In our 3D bronchial mucosa model, which integrates epithelial and stromal components, such non-lytic vesicle-mediated pathways could potentially allow bacteria egress from infected epithelial cells to infect cells located in deeper tissue layers while preserving mucosal integrity.

Consistent with this, our coculture experiments demonstrated that epithelial-derived *B. abortus* can infect adjacent lung fibroblasts. This identifies fibroblasts as potential secondary replication niches of *Brucella* within the respiratory mucosa and suggests that, in the context of a respiratory infection, bacteria released from the apical epithelium may infect fibroblasts located in the contiguous stromal compartments.

Although our observations strongly support a non-lytic release of *B. abortus* in this airway tissue context, future studies will be required to delineate the precise vesicular route(s) engaged during egress in the 3D model.

Our results also show that the bronchial mucosa increases its colagenase/gelatinase (MMP) activity in response to *B. abortus* infection. Such increased activity may result in the degradation of components of the basement membrane and the extracellular matrix, favoring the passage of *B. abortus* present in the 3D model to the basolateral compartment. In our in vitro model the bacteria detected in the basolateral CM only derives from the initial inoculum that infected the bronchial epithelium at time 0 since non-adhered or non-internalized bacteria were eliminated by washing and gentamicin treatment after the infection, and the compartment returned to ALI conditions.

In vivo, some bacteria may remain in the bronchial lumen for longer times than those used in the in vitro infection. In this situation, the increased MMP activity may favor *B. abortus* access from the lumen to the underlying tissues not only by degrading the extracellular matrix but also by altering the barrier function of the bronchial epithelium [40]. Nevertheless, this hypothesis must be further studied.

In response to infection, the bronchial mucosa secretes proinflammatory cytokines and chemokines, which together may contribute to the recruitment of neutrophils and monocytes to the site of infection [41]. It has been previously reported that non-polarized monolayers of alveolar and bronchial epithelial cells secrete IL-8 and/or MCP-1 in response to infection with *B. abortus* smooth or rough strains [17]. In the present study, the 3D bronchial model secreted IL-6, IL-8 and MCP-1 after *B. abortus* infection to a larger extent as compared to infected monolayers of polarized (this study) or non-polarized (previous studies) bronchial epithelium. We focused on these proinflammatory mediators given our previous results using monocultures of lung epithelial cells. Future studies may elucidate whether additional cytokines are released by this bronchial model in response to *Brucella*. The kinetics of cytokine secretion did not seem to relate to the kinetics of infection, as cytokine levels were highest at the time of first detection of bacteria in basolateral medium (5 days p.i.), but declined later when CFUs in either cells or medium continued to increase. To elucidate the role of each cell type in the immune response of the 3D model to *B. abortus* infection, we infected bronchial cells and fibroblasts separately. In agreement with previous studies in non-polarized bronchial epithelium [17], the polarized epithelium released IL-6 and IL-8 in response to the infection. While fibroblasts were initially thought to be devoid of immune functions [42], new studies revealed that they are sentinels that produce inflammatory mediators in response to microorganisms [43]. In particular, MRC-5 lung fibroblasts are sensitive to viral infection, and it has been shown that they release proinflammatory and anti-inflammatory cytokines, Th1- and Th2-associated cytokines, and several chemoattractans after infection with parainfluenza virus or respiratory syncytial virus [44,45]. The secretion of these immune factors involved the Akt, IκB kinase, p38 MAPK, and/or ERK1/2 signaling pathways. In addition, it has been shown that primary human lung fibroblasts produce IL-6 after infection with *Streptoccocus pneumoniae* and *Pseudomonas aeruginosa* [46]. In agreement with these studies on viral and bacterial infections, we evidence for the first time that *B. abortus* can invade and replicate in human lung fibroblasts, and that these cells respond by secreting proinflammatory cytokines (IL-6) and chemokines (IL-8 and MCP-1). These responses were mediated by NF-kB, p38 MAPK and/or PI3K pathways. Fibroblasts play a crucial role in regulation and degradation of the extracellular matrix [43]. Our findings show that these cells produce gelatinase/collagenase activity in response to *B. abortus* infection, which suggests that fibroblasts have an important role in the increase in such activity in the *Brucella*-infected 3D bronchial model.

In the context of the bronchial respiratory unit, not only fibroblasts but also epithelial cells may contribute to a proinflammatory response against *B. abortus* infection. Elucidating the communication between bronchial epithelium and lung fibroblasts is crucial for a better understanding of how the immune response to pathogens is orchestrated in the bronchial respiratory unit. Previous reports evidenced a cross-talk between these cell types, such as the release of soluble factors (IL-6, IL-8, MCP-1 and GM-CSF) by primary human lung fibroblasts in response to IL-1α present in conditioned media from damaged bronchial epithelial cells, as well as the synergistic induction of IL-6 by MRC-5 fibroblasts co-cultured with rhinovirus-infected primary human bronchial epithelial cells [47,48]. To our knowledge, this is the first study that demonstrates an interaction between pulmonary epithelial cells and lung fibroblasts in response to *B. abortus* infection through stimulation of each cell type by soluble factors released from the other cell type resulting in an increased production of IL-6, IL-8 and/or MCP-1. This cross-talk would lead to an amplification of the local innate immune response to the pathogen since both bronchial epithelial cells and lung fibroblasts would produce inflammatory mediators not only in response to *B. abortus* infection or stimulation with its antigens but also in response to factors produced by infected adjacent cells. This preliminary characterization should be followed by further studies to evaluate other proinflammatory mediators potentially involved in this cellular cross-talk in the context of *Brucella* infection.

## 5. Conclusions

In summary, our results demonstrate that *B. abortus* can infect and replicate within a human bronchial mucosa model and be released from the tissue without causing overt damage. In this context, the tissue mounts an innate response characterized by the secretion of IL-6, IL-8, MCP-1 and MMPs, likely involving reciprocal signaling between bronchial epithelial cells and lung fibroblasts. Although this response reflects active sensing of the pathogen by the airway mucosa, it appears insufficient to control the infection and tends to diminish at later stages, allowing prolonged intracellular persistence and the subsequent release of viable bacteria to the basolateral compartment. Importantly, while the 3D bronchial mucosa model used in this study does not include professional immune cells such as macrophages, neutrophils or lymphocytes, it enables the analysis of early epithelial–stromal interactions at the airway barrier. In this simplified but biologically relevant context, our findings indicate that the bronchial mucosa itself may constitute a permissive niche for *Brucella* persistence and dissemination. Future studies using complex 3D cultures or organoids or lung-on-chip models including immune cells may help to evaluate the contribution of the latter to the bronchial response to *Brucella* infection.

## Data Availability

Dataset available on request from the authors.

## Supplementary Materials

The following supporting information can be downloaded at: https://www.mdpi.com/article/10.3390/tropicalmed11030078/s1, Figure S1 : Representative cryosection (8 µm) of the mock-infected bronchial tissue model at day 5 of follow-up. E-cadherin (red) delineates the epithelial cell borders and DAPI (blue) labels cell nuclei. Images were acquired on a Zeiss LSM 710 microscope equipped with a Plan-Apochromat 40x/1.3 NA objective.
