# Supplementary figures and images for: A 3D Organotypic Human Bronchial Model Reveals Persistent Infection and Modulated Inflammatory Response when Exposed to Brucella abortus
Source: Trop Med Infect Dis. 2026 Mar 10;11(3):78. doi: 10.3390/tropicalmed11030078 (PMC13030714; doi:10.3390/tropicalmed11030078)

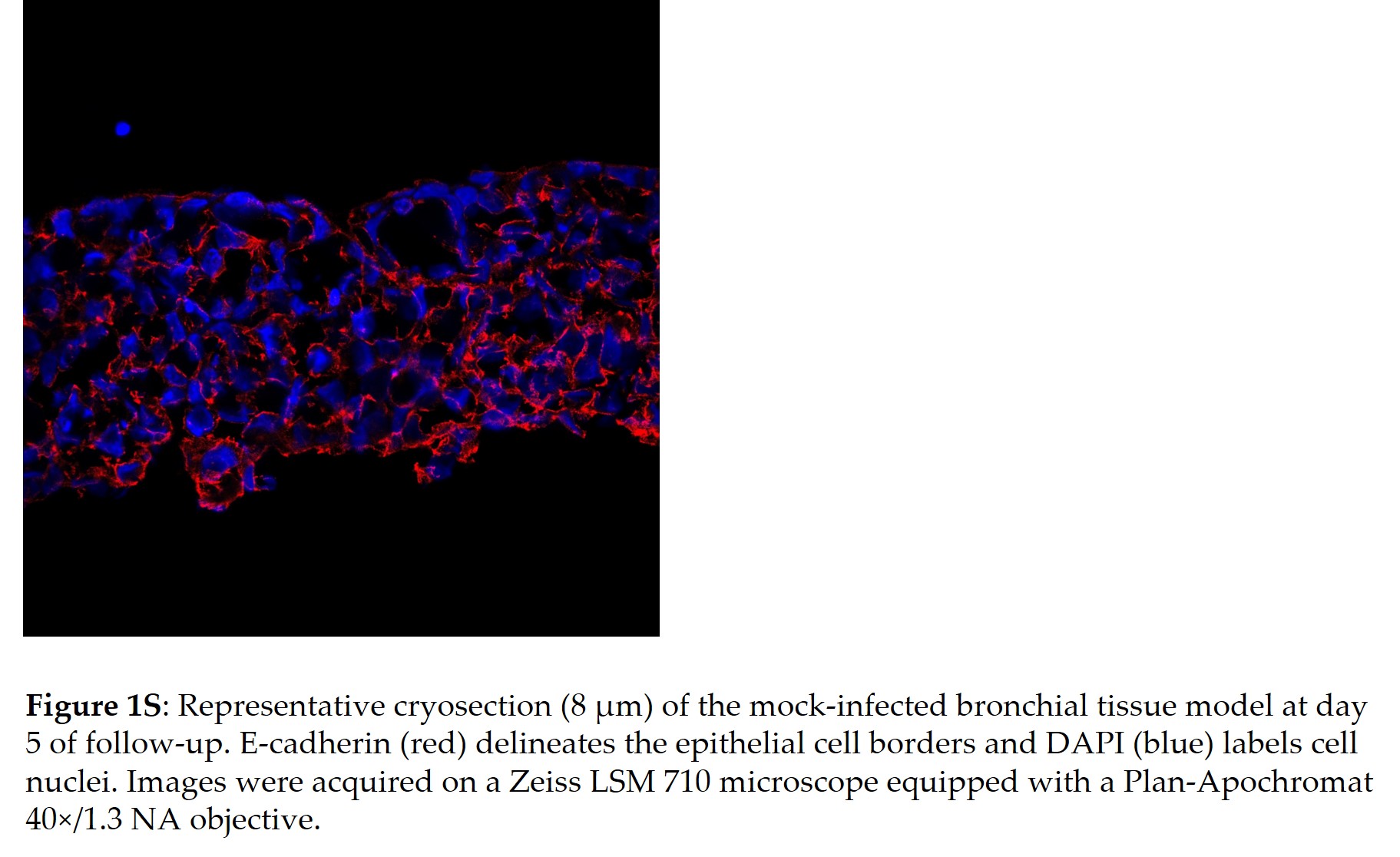

Supplement: Supplementary file 1 [file tropicalmed-11-00078-s001.zip › tropicalmed-4089598-supplementary.jpg]
